# Age-related differences in striatal dopamine D1 receptors mediate subjective drug effects

**DOI:** 10.1172/JCI164799

**Published:** 2023-01-03

**Authors:** Peter Manza, Ehsan Shokri-Kojori, Şükrü Barış Demiral, Rui Zhang, Evan Dennis, Allison Johnson, Leah Vines, Diana Sotelo, Dardo Tomasi, Gene-Jack Wang, Nora D. Volkow

**Affiliations:** National Institute on Alcohol Abuse and Alcoholism, NIH, Bethesda, Maryland, USA.

**Keywords:** Neuroscience, Addiction, Neuroimaging, Psychiatric diseases

**To the Editor:** The brain’s dopamine system is critical for cognition, reward, and motivation. It plays a crucial role in the reinforcing effects of drugs with addiction potential as well as in some of their therapeutic effects (e.g., stimulants, such as methylphenidate [MP]) (1). Importantly, aging is associated with declines in key proteins involved in dopamine signaling, including D1- and D2-like dopamine receptors (D1Rs and D2Rs) and dopamine transporters (2), and there is some evidence that elderly individuals are less sensitive to the subjective effects of stimulant drugs than young adults (3). This opens the possibility that age-related declines in dopamine signaling in brain reward regions affect sensitivity to the positive reinforcing effects of drugs, a key risk factor for the development of a substance use disorder (SUD) (4). This would help explain why substance use and SUD rates are much lower among elderly individuals compared with those among younger adults (5). If specific brain markers of dopamine signaling are tied to aging and drug effects, then this information could be leveraged to better understand individual differences in vulnerability to substance misuse and SUD.

Using PET imaging, we collected measures of D1Rs and D2Rs and estimates of MP-induced dopamine increases to study the effect of age on the subjective experience of MP in healthy adults. We hypothesized that striatal D1R would show a stronger association with subjective MP effects than striatal D2 and that striatal D1R availability would mediate the link between age and subjective MP effects.

Thirty-six healthy adults (23 men and 13 women; age, 22–64 years) participated (for detailed demographics, see Supplemental Table 1; supplemental material available online with this article; https://doi.org/10.1172/JCI164799DS1). All participants provided written informed consent prior to participation. The NIH IRB approved the study.

PET scans measured D1R availability with [^11^C]NNC-112 and D2R availability with [^11^C]raclopride. All [^11^C]NNC-112 scans were conducted in a baseline state. [^11^C]raclopride scans were conducted on 2 separate days: (a) 1 hour after administration of an oral placebo pill and (b) 1 hour after administration of 60 mg oral MP to quantify dopamine increases by comparing changes in D2R availability with placebo scans (6). Before [^11^C]raclopride scans, drugs were administered in a single-blind design, and session order was counterbalanced. All participants underwent all 3 PET scans.

Periodically throughout the sessions, participants were asked to rate, on a scale of 1 to 10, several questions about their subjective experience of drug reward in response to MP. We characterized associations among D1R, D2R, and dopamine increases with age and subjective drug effects at the voxel level and at the region-of-interest (accumbens and dorsal striatum) level. We then performed mediation analysis, hypothesizing that accumbens D1R would mediate the negative association between age and subjective drug effects. For all analyses, a *P* value of less than 0.05 was considered significant. For more details, see Supplemental Methods.

There was a strong negative linear association between D1R and age that was relatively uniform throughout the striatum, whereas the D2R-age association was weaker overall, compared with the D1R-age association, and specific to the head of caudate and posterior putamen. There were no significant associations between age and MP-induced dopamine increases. We also observed a positive linear association between D1R and “feeling drug effects” (peak in accumbens; Montreal Neurological Institute coordinates [*x* = 14, *y* = 14, *z* = –6; peak *t* = 3.55). There were no significant associations between feeling drug effects and D2R availability or MP-induced dopamine increases (Figure 1A).

In region-of-interest robust correlation analysis, we observed significant associations with age and D1R, both in dorsal striatum (*r* = –0.52, Bonferroni’s corrected *P* [*P*_bonf_] = 0.0028) and accumbens (*r* = –0.50, *P*_bonf_ = 0.004). However, these associations with age were not significant for D2R and MP-induced dopamine increases. We also observed significant associations with feeling drug effects and D1R, both in dorsal striatum (*r* = 0.466, *P*_bonf_ = 0.008) and accumbens (*r* = 0.599, *P*_bonf_ = 0.0002). However, associations with feeling drug effects were not significant for D2R and MP-induced dopamine increases (for associations with other brain regions, see Supplemental Table 2).

The subjective effects of 60 mg oral MP relative to placebo were negatively associated with age, as we hypothesized (Figure 1B; *r* = –0.37, *P* = 0.026).

A Fisher’s *z* test for comparing correlations from dependent samples revealed that the association of accumbens D1R with subjective effects was significantly stronger than the D2R–subjective effects association (*z* = 3.699, *P* < 0.001; Figure 1C). Accumbens D1R mediated the link between older age and lower subjective drug effects (indirect effect coefficient = –2.12; 97.5% CI [–6.54, –0.37]; *P* = 0.004; Figure 1D).

In sum, declines in accumbens D1R availability (but not D2R availability or MP-induced dopamine increases) may explain why people feel stimulant drug effects less strongly as they age, providing a possible neural mechanism for the lower prevalence of stimulant use disorders in elderly individuals compared with that in young adults (5).

## Supplementary Material

Supplemental data

## Figures and Tables

**Figure 1:**
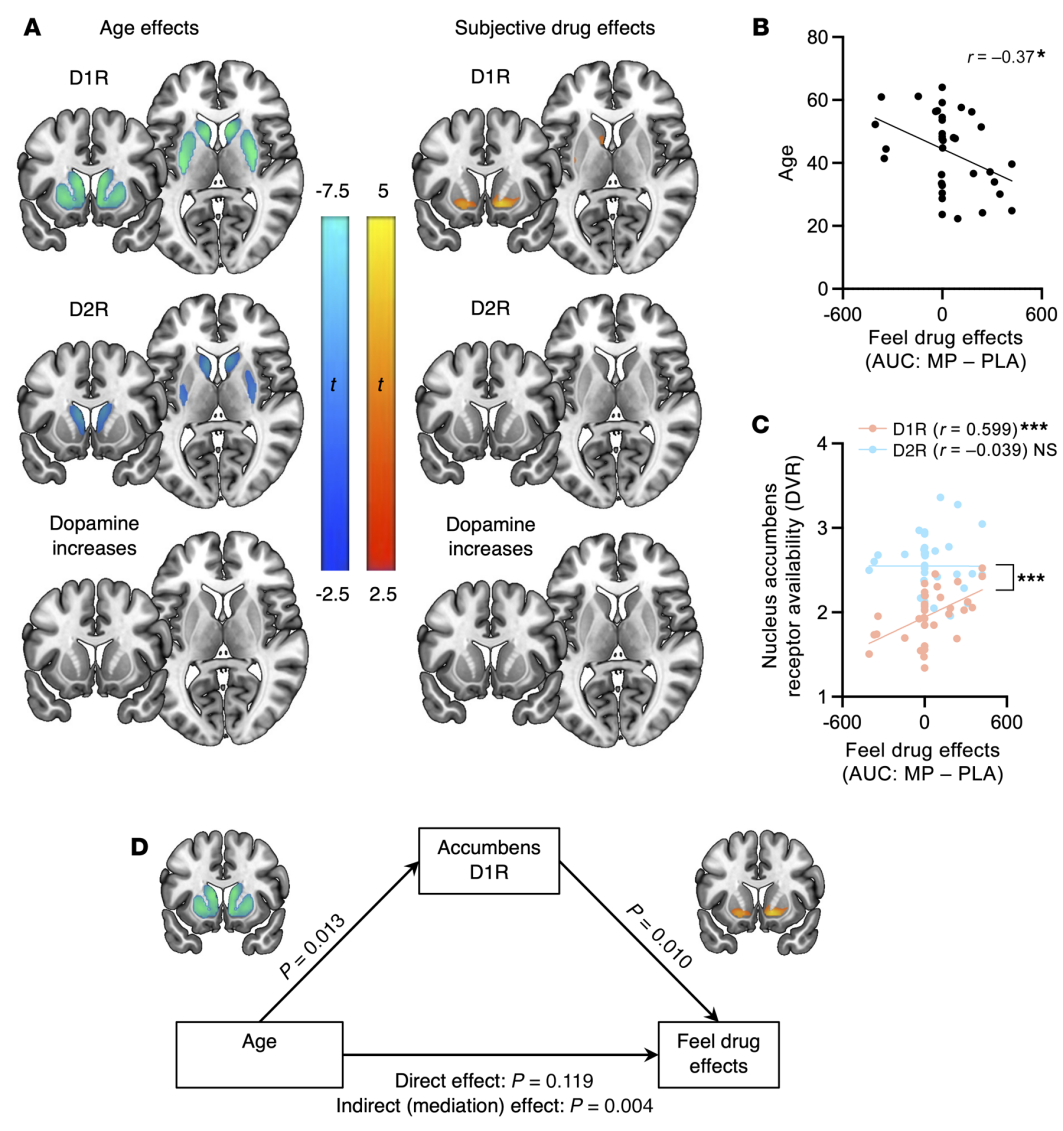
Associations among markers of brain dopamine system function, age, and subjective drug effects. (**A**) Regression plots depicting the association of age and subjective drug effects with striatal dopamine D1-like dopamine receptor (D1R), D2R, and methylphenidate-induced (MP-induced) dopamine increases. All analyses were controlled for BMI and sex. (**B**) Negative association between subjective drug effects and age. To quantify total effects of the drug across the session, we calculated area under the curve (AUC) for all time points (every 5 minutes, from –5 minutes to 120 minutes after administration of MP) and subtracted the AUC between the MP and placebo (PLA) sessions to get one estimate of the cumulative drug effects per participant. Thus, a negative value for “feel drug effects” indicates a greater subjective response to PLA than MP. (**C**) A selective role for D1R but not D2R in the subjective effects of MP. Nucleus accumbens D1R but not D2R availability was significantly associated with subjective drug effects. The difference in regression slopes was significant (*z* = 3.699, *P* < 0.001). (**D**) D1R availability in the nucleus accumbens mediates the negative association between age and subjective drug effects. DVR, distribution volume ratio. *P < 0.05; ***P < 0.001.

## Version 1 11/10/2022

In-Press Preview

## Version 2 01/03/2023

Electronic publication
