# Supplementary material for: Age-related differences in striatal dopamine D1 receptors mediate subjective drug effects
Source: J Clin Invest. 2023 Jan 3;133(1):e164799. doi: 10.1172/JCI164799 (PMC9797329; doi:10.1172/JCI164799)
Supplement: Supplemental data [file jci-133-164799-s110.pdf]

Supplementary Material for:

Age-related differences in striatal dopamine D1 receptors mediate subjective drug effects

Trial Registration. ClinicalTrials.gov: NCT03190954.

Funding. National Institute on Alcohol Abuse and Alcoholism (ZIAAA000550).

Acknowledgements. We thank Michele Vera-Yonga, Veronica Ramirez, Jamie Burns, Christopher Kure Liu, Dani Kroll, Dana Feldman, Karen Torres, Christopher Wong, Amna Zehra, Lori Talagala, Myke Vandine and Minoo McFarland for their contributions. This work was accomplished with support from the National Institute on Alcohol Abuse and Alcoholism ZIAAA000550).

Author Contributions:
NDV conceived of and designed the study. PM, ESK, RZ, ED, AJ, LV, and DS acquired the data. PM, ESK, SBD, RZ, DT, NDV performed data analysis and interpretation. PM wrote the first draft of the manuscript. All authors revised and gave final approval to the manuscript.

METHODS AND MATERIALS

Participants. Participants were excluded if they had a history of substance abuse or dependence (other than nicotine) or a history of psychiatric disorder, neurological disease, medical conditions that may alter cerebral function (i.e., cardiovascular, endocrinological, oncological, or autoimmune diseases), current use of prescribed or over-the-counter medications, and/or head trauma with loss of consciousness of > 30 min. Nineteen of 36 participants reported drinking alcohol. Among those drinking alcohol, 10 reported only drinking "socially" or once a month maximum, five reported drinking twice weekly, and four reported drinking four times weekly. In addition, all participants reported no prior exposure to cocaine, amphetamine, methamphetamine, cannabis, or opiates. Portions of the PET data were analyzed differently in prior publications (1–3). However, the main analyses and findings described in this manuscript are original.

For each individual, studies were conducted on one of two scanners: a high-resolution research tomography (HRRT) scanner (n = 17; 7 female; Siemens AG; Germany) or a Biograph PET/CT scanner (n = 19; 6 females; Siemens AG; Germany). The use of two different scanners was necessary due to scheduling limitations at our site. The methods for correcting differences between scanners are described in the PET analysis section below. All [^{11}C]NNC-112 scans were conducted at 10AM, in a baseline state, without any drug manipulation. [^{11}C]raclopride scans were conducted on two separate days: once 1 h after administration of an oral placebo pill and once 1 h after administration of 60 mg oral MP, which was done to quantify dopamine increases by comparing changes in D2R availability with the placebo scans. The [^{11}C]raclopride scans were single blind and the order of sessions was counterbalanced across all participants, as well as within

scanners (for HRRT, MP was administered on the first and second day in 9 and 8 participants, respectively, and for PET/CT, MP was administered on the first and second day in 10 and 9 participants, respectively) and across ages (average age of subjects receiving MP on the first day = 42.85, and second day = 44.47; two-sample *t*-test: $p = .70$). Raclopride scans were conducted at the same time of day (1 PM) and in the same scanner for a given participant.

For [^{11}C]NNC-112, twenty-one sequential dynamic emission scans were started immediately after a maximum injection of 555 MBq (Specific activity at time of injection = 4794 ± 2483 mCi/umole; dose mass = 1.41 ± .88 ug); scans were obtained for a total of 90 minutes to measure the radiotracer's uptake, plateau and clearance (time activity curves), which are needed to quantify receptor availability. For [^{11}C]raclopride, twenty-two sequential dynamic emission scans were started immediately after a maximum injection of 370 MBq (for placebo scans: specific activity at time of injection = 5466 ± 2895 mCi/umole; mass = .80 ± .42 ug; and for MP scans: specific activity at time of injection = 5804 ± 2349 mCi/umole; mass = .76 ± .48 ug); scans were obtained for a total of 60 minutes. Dynamic emission scan images were evaluated before analyses to ensure that motion artifacts or misplacements were not included.

PET analysis

PET images were co-registered to the high-resolution MRI T1 and T2 structural images. We used the minimal preprocessing pipelines of the Human Connectome Project for the spatial normalization to the stereotactic MNI space of the structural MRI and subsequently the PET scans (4). Differences in geometry and PSF between cameras (PET/CT = 4 mm FWHM; HRRT = 2.7 mm FWHM) resulted in systematic voxelwise differences in signal intensity between PET/CT and HRRT images. To correct for these scanner-specific scaling effects and harmonize the data we

used a voxelwise approach based on grand-mean scaling. We used an updated version of the ComBat Harmonization technique implemented in the ENIGMA study(5). Originally proposed by Johnson et al. (6) and implemented in the surrogate variable analysis (sva) package in R (7), ComBat uses an Empirical Bayes framework to estimate the distribution scanner effects. It was shown to be superior to other harmonization methods for varieties of data types including DTI (8) and cortical thickness (9). We conducted ComBat separately for each tracer for the PET measure of interest (i.e., receptor availability) to harmonize the data across scanners. ComBat was applied directly to the final distribution volume ratio (DVR) images (see *D1R/D2R availability: Striatum* section below). For [^{11}C]raclopride measures, since we had placebo and methylphenidate treatments, we used drug, age, sex (male/female) and race (4 groups; white, black, Asian and Native American) as covariates in the model. For [^{11}C]NNC-112 measures, since there was no drug manipulation, we used only age, sex, and race as covariates.

FreeSurfer version 5.3.0 (http://surfer.nmr.mgh.harvard.edu) was used to automatically segment the anatomical MRI scans using the Desikan atlas (10), which provided bilateral nucleus accumbens, caudate, putamen, and cerebellum regions of interest.

D1R/D2R availability: Striatum. Time–activity curves in the dorsal striatum (caudate and putamen), accumbens, and cerebellum were used to obtain the distribution volume ratios (DVR) using a Logan reference tissue model (11, 12). The accumbens-to-cerebellum and the dorsal striatum-to-cerebellum distribution volume ratios correspond to BPnd+1, which was used to quantify D1R and D2R receptor availability. We averaged the values for caudate and putamen to create one 'dorsal striatum' region, since caudate and putamen BPnd are highly correlated with one another (across participants, $r \approx .9$). We also used the D2R availability estimates to compute 'dopamine increases' based on previous work:

$$Dopamine\ Increases = \frac{D2Rplacebo - D2Rmethylphenidate}{D2Rplacebo} \tag{1}$$

Subjective Drug Effects: Participants were asked to rate, on a scale of 1 to 10, several questions regarding drug effects, including the following related to drug reward: 1) "do you feel drug effects?" 2) "do you like the effects?" 3) "do you want more of what you received?" 4) "do you feel high?". These questions were asked every five minutes, starting five minutes prior to MP and placebo administration, and continuing up to two hours post-drug administration. Across participants, responses to these questions were very similar: the average pairwise correlation coefficient across questions was $r = 0.77$. Therefore, to reduce redundant analyses, we chose to continue only with the responses to "do you feel drug effects?", for which the greatest number of participants endorsed any value greater than one, the minimum (24 of 36 participants) and which was therefore most suitable for regression analysis. "Feeling drug effects" was thus used as a proxy of subjective drug reward, and for the primary mediation analysis (see *Statistics* section below) we confirmed that results were similar for the other questions. To get a single estimate of the total subjective response to the drug over the two-hour period, we calculated the area under the curve of their responses at all timepoints using GraphPad Prism version 9.4.0. We then took the difference of the AUC across the two sessions (MP minus placebo) as the main outcome measure of the subjective effects of MP.

Statistics

For descriptive purposes, we first sought to characterize the associations between D1R, D2R, dopamine increases and age/subjective drug effects at the voxel level. We performed

multiple regression, with age or 'feeling drug effects' ratings as the predictor and sex and BMI as covariates, over all striatal voxels, using the SPM12 toolbox in MATLAB.

We then tested whether age is negatively associated with the subjective effects of MP. We also tested if D1R, D2R, and dopamine increases were significantly associated with subjective drug effects (each analysis was performed separately for dorsal striatum and accumbens; results were Bonferroni-corrected for two regions of interest). For these analyses we performed 'Shepherd's Pi' correlations(13), which are robust to the presence of outliers. We hypothesized stronger associations would be observed with D1R than D2R, based on postmortem studies showing a stronger negative association of D1R with age than D2R (14, 15) and that D1R, particularly in the accumbens, is critical for drug reward (16). We then performed 'causal' mediation analysis, hypothesizing that accumbens D1R would mediate the negative association between age and subjective drug effects. Although in one prior study there were hemispheric differences in D1R (17), in our sample there was a strong correspondence in striatal D1R availability between the left and right hemispheres ($r = .83$ for dorsal striatum and $r = .67$ for accumbens), and we therefore averaged estimates across left and right hemispheres. We nonetheless confirmed that primary findings were significant for both left and right hemispheres individually. Correlation and mediation analyses were performed in the Pingouin package in Python 3.9 (18). As a control analysis, we also tested whether age and subjective drug effects were significantly associated with peripheral measures of MP effects: MP-induced increases in heart rate (calculated using the same AUC approach as the subjective drug effects) and plasma concentrations of MP (as detailed in our prior work(3); this data was available for 28 of 36 participants).

References

1. Manza P et al. Cortical D1 and D2 dopamine receptor availability modulate methylphenidate-induced changes in brain activity and functional connectivity. *Commun Biol* 2022;5(1):1–9.

2. Zhang R et al. Dopamine D1 and D2 receptors are distinctly associated with rest-activity rhythms and drug reward [Internet]. *Journal of Clinical Investigation* 2021;131(18). doi:10.1172/JCI149722

3. Manza P et al. Sex differences in methylphenidate-induced dopamine increases in ventral striatum. *Mol Psychiatry* 2022;27(2):939–946.

4. Glasser MF et al. The minimal preprocessing pipelines for the Human Connectome Project. *NeuroImage* 2013;80:105–124.

5. Radua J et al. Increased power by harmonizing structural MRI site differences with the ComBat batch adjustment method in ENIGMA. *NeuroImage* 2020;218(May). doi:10.1016/j.neuroimage.2020.116956

6. Johnson WE, Li C, Rabinovic A. Adjusting batch effects in microarray expression data using empirical Bayes methods. *Biostatistics* 2007;8(1):118–127.

7. Leek JT, Johnson WE, Parker HS, Jaffe AE, Storey JD. The SVA package for removing batch effects and other unwanted variation in high-throughput experiments. *Bioinformatics* 2012;28(6):882–883.

8. Fortin JP et al. Harmonization of multi-site diffusion tensor imaging data. *NeuroImage* 2017;161(July):149–170.

9. Fortin JP et al. Harmonization of cortical thickness measurements across scanners and sites. *NeuroImage* 2018;167(June 2017):104–120.

10. Desikan RS et al. An automated labeling system for subdividing the human cerebral cortex on MRI scans into gyral based regions of interest. *NeuroImage* 2006;31(3):968–980.

11. Logan J et al. Graphical Analysis of Reversible Radioligand Binding from Time—Activity Measurements Applied to [N - 11 C-Methyl]-(−)-Cocaine PET Studies in Human Subjects. *Journal of Cerebral Blood Flow & Metabolism* 1990;10(5):740–747.

12. Logan J et al. Distribution Volume Ratios without Blood Sampling from Graphical Analysis of PET Data. *Journal of Cerebral Blood Flow & Metabolism* 1996;16(5):834–840.

13. Schwarzkopf DS, De Haas B, Rees G. Better Ways to Improve Standards in Brain-Behavior Correlation Analysis [Internet]. *Front. Hum. Neurosci.* 2012;6. doi:10.3389/fnhum.2012.00200

14. Rinne JO, Lönnberg P, Marjamäki P. Age-dependent decline in human brain dopamine D1 and D2 receptors. *Brain Research* 1990;508(2):349–352.

15. Seeman P et al. Human brain dopamine receptors in children and aging adults. *Synapse* 1987;1(5):399–404.

16. Park K, Volkow ND, Pan Y, Du C. Chronic cocaine dampens dopamine signaling during cocaine intoxication and unbalances D1 over D2 receptor signaling. *Journal of Neuroscience* 2013;33(40):15827–15836.

17. Cannon DM et al. Dopamine Type-1 Receptor Binding in Major Depressive Disorder Assessed Using Positron Emission Tomography and [11C]NNC-112. *Neuropsychopharmacology* 2009;34(5):1277–1287.

18. Vallat R. Pingouin: statistics in Python. *Journal of Open Source Software* 2018;3(31):1026.

Supplemental Table 1. **Demographics and participant characteristics, by PET scanner.** Note: BMI = Body-Mass Index; IQ = Intelligence Quotient.; SD = Standard Deviation. †, χ^2 test-statistic

		Scanner 1: HRRT (n = 17)	Scanner 2: PET/CT (n = 19)	$t_{(df)}$, p
**Age**	Min-Max	33-64	22-60	
	Mean ± SD	48.41 ± 9.60	39.31 ± 12.83	$2.39_{(34)}$, .023
**Sex**	n, Female (%)	7 (41)	6 (32)	$.062_{(1)}$, .801 †
**BMI**	Min-Max	21-39	21-33	
	Mean ± SD	27.19 ± 5.09	28.24 ± 3.28	$0.74_{(34)}$, .463
**IQ**	Min-Max	79-139	97-129	
	Mean ± SD	122.35 ± 15.80	110.05 ± 11.58	$2.64_{(34)}$, .011
**Race**	n, White (%)	11 (65)	5 (26)	$7.16_{(3)}$, .067 †
	n, Black (%)	6 (35)	10 (53)	
	n, Asian (%)	0 (0)	2 (11)	
	n, Native American (%)	0 (0)	2 (11)	

Supplemental Table 2. **Exstrastriatal dopamine D1 receptor binding and associations with age and subjective drug effects.** All regions were defined using the Harvard-Oxford Atlas. Note: DVR = Distribution volume ratio.

	DVR	Correlation: Age (*r*)	Correlation: Feeling Drug Effects (*r*)
**CORTICAL REGIONS**			
Frontal Pole	0.169	-0.737	0.442
Insular Cortex	0.475	-0.855	0.442
Superior Frontal Gyrus	0.118	-0.721	0.292
Middle Frontal Gyrus	0.140	-0.715	0.480
Inferior Frontal Gyrus pars triangularis	0.101	-0.543	0.547
Inferior Frontal Gyrus pars opercularis	0.216	-0.752	0.507
Precentral Gyrus	0.075	-0.690	0.471
Temporal Pole	0.128	-0.732	0.546
Superior Temporal Gyrus anterior division	0.261	-0.697	0.464
Superior Temporal Gyrus posterior division	0.244	-0.690	0.487
Middle Temporal Gyrus anterior division	0.163	-0.543	0.483
Middle Temporal Gyrus posterior division	0.243	-0.649	0.540
Middle Temporal Gyrus temporooccipital part	0.230	-0.616	0.475
Inferior Temporal Gyrus anterior division	0.189	-0.503	0.494
Inferior Temporal Gyrus posterior division	0.223	-0.508	0.494
Inferior Temporal Gyrus temporooccipital part	0.266	-0.683	0.470
Postcentral Gyrus	0.048	-0.619	0.446
Superior Parietal Lobule	0.042	-0.576	0.381
Supramarginal Gyrus anterior division	0.104	-0.607	0.414
Supramarginal Gyrus posterior division	0.135	-0.660	0.463
Angular Gyrus	0.264	-0.721	0.414
Lateral Occipital Cortex superior division	0.135	-0.706	0.473
Lateral Occipital Cortex inferior division	0.173	-0.555	0.493
Intracalcarine Cortex	0.426	-0.656	0.400
Frontal Medial Cortex	0.442	-0.757	0.424
Supplementary Motor Cortex	0.193	-0.708	0.358
Subcallosal Cortex	0.416	-0.816	0.471
Paracingulate Gyrus	0.394	-0.843	0.452
Cingulate Gyrus anterior division	0.316	-0.856	0.487
Cingulate Gyrus posterior division	0.349	-0.801	0.427
Precuneous Cortex	0.362	-0.715	0.435
Cuneal Cortex	0.324	-0.581	0.370

Frontal Orbital Cortex	0.260	-0.768	0.537
Parahippocampal Gyrus anterior division	0.008	-0.550	0.378
Parahippocampal Gyrus posterior division	0.080	-0.676	0.528
Lingual Gyrus	0.294	-0.710	0.618
Temporal Fusiform Cortex anterior division	0.208	-0.557	0.373
Temporal Fusiform Cortex posterior division	0.246	-0.689	0.471
Temporal Occipital Fusiform Cortex	0.293	-0.739	0.518
Occipital Fusiform Gyrus	0.310	-0.623	0.610
Frontal Operculum Cortex	0.406	-0.791	0.433
Central Opercular Cortex	0.376	-0.830	0.465
Parietal Operculum Cortex	0.345	-0.768	0.379
Planum Polare	0.297	-0.812	0.368
Heschl's Gyrus	0.417	-0.845	0.414
Planum Temporale	0.352	-0.758	0.382
Supracalcarine Cortex	0.505	-0.668	0.388
Occipital Pole	0.044	-0.332	0.404
**SUBCORTICAL REGIONS**			
Thalamus	0.121	-0.437	0.227
Pallidum	0.645	-0.624	0.501
Hippocampus	0.107	-0.627	0.403
Amygdala	0.186	-0.645	0.496
